# The Possible Importance of Glutamine Supplementation to Mood and Cognition in Hypoxia from High Altitude

**DOI:** 10.3390/nu12123627

**Published:** 2020-11-25

**Authors:** MVL Dos Santos Quaresma, WYG Souza, VA Lemos, AV Caris, RV Thomatieli-Santos

**Affiliations:** 1 Department of Nutrition, Centro Universitário São Camilo, São Paulo 04263-200, Brazil; marcus.santos.nutri@gmail.com (M.D.S.Q.); alinecaris@hotmail.com (A.C.); 2 Department of Bioscience, Universidade Federal de São Paulo, São Paulo 11015-020, Brazil; rvca5@gmail.com (W.S.); aquino.lemos@terra.com.br (V.L.); 3 Department of Psychobiology, Universidade Federal de São Paulo, São Paulo 04023-062, Brazil

**Keywords:** hypoxia, mood, cognition, inflammation, glutamine

## Abstract

Hypoxia induced by low O_2_ pressure is responsible for several physiological and behavioral alterations. Changes in physiological systems are frequent, including inflammation and psychobiological declines such as mood and cognition worsening, resulting in increased reaction time, difficulty solving problems, reduced memory and concentration. The paper discusses the possible relationship between glutamine supplementation and worsening cognition mediated by inflammation induced by high altitude hypoxia. The paper is a narrative literature review conducted to verify the effects of glutamine supplementation on psychobiological aspects. We searched MEDLINE/PubMed and Web of Science databases and gray literature by Google Scholar for English articles. Mechanistic pathways mediated by glutamine suggest potential positive effects of its supplementation on mood and cognition, mainly its potential effect on inflammation. However, clinical studies are scarce, making any conclusions impossible. Although glutamine plays an important role and seems to mitigate inflammation, clinical studies should test this hypothesis, which will contribute to a better mood and cognition state for several people who suffer from problems mediated by hypoxia.

## 1. Introduction

High altitude regions are frequently visited for several reasons, such as sports training, tourism, scientific research, work or military operations [1]. In South America between 2003 and 2004, 6994 people visited the Aconcagua Provincial Park in the Argentine Andes, with an altitude of approximately 6960 m. Of this total, 60%, including climbing activities. Despite the beautiful landscapes, high altitude regions pose risks to human health. In the Himalayan region, from 1950 to 2006, 784 deaths were recorded at altitudes above 6000 m. The adverse effects observed at high altitudes can be triggered by the partial pressure of oxygen (PO_2_) compared to sea level [2].

Barometric pressure reduction leads to decreased PO_2_ per unit volume, which reduces the amount of oxygen (O_2_) available in the blood and tissues, characterizing hypoxia. In these conditions, significant physiological alterations can occur, such as increased pro-inflammatory cytokines and C-reactive protein [3]. Therefore, strategies that can mitigate the effects of hypoxia at high altitudes should be studied, including glutamine, which is the most abundant and versatile amino acid in the human body and has high anti-inflammatory action [4]. Glutamine is a crucial energy fuel for rapidly growing cells (mostly immune and enterocytes cells), the precursor of glucose, proteins and nucleic acids, having diverse roles in the organism, being key to the synthesis of cytokines, hormones, acid-base balance, transport of ammonia and cell proliferation [5]. Glutamine supplementation has been studied to reduce the risk of upper respiratory tract infections (URTI) after long-term exercise (i.e., marathon), thus improving immunocompetence in normoxia [6] and hypoxia [7,8,9]. The high blood glutamine availability can interfere with central glutamate synthesis, an excitatory neurotransmitter that stimulates ventilation, increasing O_2_ saturation (SaO_2_%) [10]. Glutamine can positively affect the function of enterocytes and immune cells resident in the intestine and improve the defense mechanism. Moreover, it can serve as an energetic substrate for gut microorganisms, reducing the inflammatory process mediated by increased gut permeability [11]. Finally, glutamine could directly modify neurotransmitter actions in the brain, affecting mood and cognition [12].

This review has two objectives: (1) to discuss the possible impacts of inflammation on worsening mood and cognition reported by people exposed to high altitude regions and (2) to examine the role of glutamine supplementation on mood and cognition in the hypoxia condition. Several studies have demonstrated the relation between glutamine and anti-inflammatory effects by showing their influence on inflammatory signaling pathways, including the nuclear factor κB (NF-κB) and signal transducer and activator of transcription (STAT) [13]. Other studies show that glutamine can modulate aspects of the central nervous system and improve psychobiological aspects [12,14,15]. For example, glutamine supplementation can reduce subjective fatigue and ratings of perceived exertion during demanding tasks [13,16]. Thus, we propose that glutamine supplementation may reduce cognition worsening mediated by inflammation induced by high altitude hypoxia.

### Study Type and Search Strategy

A narrative literature review was conducted to speculate and elucidate the potential effects of glutamine supplementation on psychobiological aspects. We searched in MEDLINE/PubMed and Web of Science databases and gray literature by Google Scholar for English published articles until July 2020. Multiple search MeSH terms, entry terms, and keywords combinations were used, verified in Table 1. All the descriptors searched using the Boolean operators OR and AND to obtain a full search. Two hundred and forty-seven articles of potential interest were found. After reading the abstracts, the 101 used to prepare the review were selected.

Considering that inflammation is determinant in the hypoxia situation, we select articles related to these topics and changes in the central nervous system. Moreover, we selected articles that analyzed the relationship between glutamine supplementation, inflammation, mood and cognition. We used animal and human studies to understand the proposed mechanisms and the clinical effects of glutamine supplementation on psychobiological aspects.

## 2. Hypoxia

An increase in altitude is associated with an exponential decrease in barometric pressure and partial reduction in the O_2_ per unit volume. The concentration of O_2_ required for the human organism to function adequately is 21%, which equals sea level. At high altitude, the decrease in oxygen available for cellular metabolism compromises the permanence and performance of the human being and presents several repercussions in the homeostasis of different biological systems [17]. Several acute physiological responses tend to minimize the deleterious effects of the low O_2_ available in different tissues, including changes in different physiological systems, such as respiratory, cardiovascular, immunological and hormonal and hematological components [18].

A fast ascent to altitudes above 2500 m leads to headaches, nausea, vomiting, gastrointestinal changes, lack of appetite, fatigue and insomnia, symptoms known as acute mountain sickness (AMS) [19]. These symptoms occur because of the inability to maintain cellular functioning since 33–50% of cerebral oxygen is for synaptic transmission. When the O_2_ is insufficient, hypoxia-inducible factor 1 (HIF-1) affects synaptic transmission in a few minutes [20], negatively influencing the tasks.

## 3. Hypoxia and Mood

The mood is considered a temporary state of pleasure or displeasure. It may present many variations in response to a particular situation and be of variable duration, including a few hours to days. In hypoxia, negative dimensions such as tension, depression, anger, fatigue and confusion may increase, while vigor decrease [20,21]. Worsening mood states can affect the performance of tasks that require a balanced emotional state. This balance is the result of the interaction of behavioral patterns, physiological changes and conscious experiences. For example, Li et al. (2000) verified the mood state in 18 male volunteers in a hypobaric chamber simulating 300 m (control), 2800 m, 3600 m and 4400 m. The researchers observed that negative mood dimensions (tension, fatigue, among others) gradually increased with high altitude, while positive mood (vigor–activity) tended to decrease [22]. Bolmont et al. (2000) investigated the mood changes of 8 climbers undergoing hypoxia using a simulated 8848 m climb in a hypobaric chamber [23]. The authors observed that mood worsened as hypoxia increased. To explain this phenomenon, the authors bet on HIF-1 released under hypoxic conditions. Shibata et al. (2013) tested the hypothesis that HIF-1 could be associated with mood disorder pathophysiology. One hundred thirty-one patients were divided into control, depressive and bipolar groups, and the results demonstrated an association between HIF-1, its target genes in peripheral blood cells and mood disorders (especially depressive symptoms) [24].

At altitude, mood alterations are more prevalent during the first or second day of exposure to the hypoxic environment. From an altitude of 3000 m, the human being can suffer mood modifications, increasing irritability, fatigue and decreased muscle strength for extended periods such as 30 days [25].

Bardwell et al. (2005) verified the effects of strenuous training on mood at altitudes of 2053 m to 3600 m for 30 days. The authors observed that the volunteers increased negative mood at the moment of practice, such as tension and confusion and worsening vigor. Ninety days after completing the training, the assessors re-performed the mood tests with the volunteers and observed that only vigor returned to baseline [26]. Mood state is affected by exposure hypoxia duration, altitude and high variability between individuals. Therefore, it is critical to elucidate the possible mechanisms for such mood state alterations since they can negatively influence the variables related to this emotional state, such as cognition, decision-making and motor skills execution.

## 4. Hypoxia and Cognition

Recent studies by our laboratory and other groups have drawn attention to hypoxia effect on cognitive aspects, comprising intellectual abilities that facilitate reasoning, perception, communication, problem-solving, learning and reaction time [27,28,29]. These intellectual abilities are fundamental to daily living, for example, for learning a stipulated task or when a driver sights an obstacle in front of them and must present a reaction before reaching it, that is, independent of the environment or the functions. These abilities can undergo considerable alterations and reduce cognitive performance by up to 50% at an altitude of 5000 m [28]. Moreover, hypoxia can interfere in health and professional performance, especially for people exposed to hypoxic environments and require many cognitive functions, such as airplane pilots, since a single fault can interfere in other people’s lives. Kryskow et al. (2016) analyzed whether the decline in cognitive function depends on the altitude level. In this study, volunteers were divided into 4 groups: 2500 m (*n* = 17), 3000 m (*n* = 12), 3500 m (*n* = 11) and, 4300 m (*n* = 17) exposed to altitude for 8 h. Simple psychomotor performance was not affected at any altitude, while complex psychomotor performance worsened in volunteers exposed to an altitude of 4300 m [30].

In addition to worsening accountability and reaction time, hypoxia can also alter short-term memory considering two critical aspects: information storage and information retrieval speed [20]. Other studies demonstrate that exposure to altitudes between 2800 and 4400 m for one hour worsens continuous recognition memory and reaction time [31].

Exposure to hypobaric hypoxia over a 4.5 h period was evaluated using several standardized AMS tests, mood and motor and cognitive performance. Twenty-three volunteers were exposed to 3 conditions: normoxia, 4200 m and 4700 m, with 48 h intervals between each altitude [32]. The authors observed that at 4700 m, all analyzed variables worsened, while at 4200 m, it was possible to observe a worsening of mood and motor and cognitive performance in both conditions.

Bijursten et al. (2010) investigated the effects of altitude on cognitive function and S-100B protein release (expressed predominantly by central nervous system cells, mainly astroglia cells). The higher S100-B in serum suggests a rupture of the blood–brain barrier (BBB). Seven volunteers climbed a mountain with an altitude of 4554 m, performed cognitive and acute mountain sickness tests and blood samples to measure the S-100B levels at five time-points. The results showed progressive increases in AMS, reduced cognitive function and increases in S-100B protein of up to 80% compared to the climb’s beginning. These results demonstrate that neuronal damage may have occurred due to the hypoxic environment in which cerebral oxygen flow is reduced [33].

Gao et al. (2015) analyzed how long-term migration to high-altitude regions affected mood and cognition and correlated them with physiological and biochemical changes [21]. For this study, 358 volunteers were divided into two groups of young males: sea level (*n* = 141) and residents at an altitude of 4500 m from 1 to 5 years (*n* = 217). Mood, cognition and sleep evaluation were carried out. The SaO_2_%, S-100B protein and brain-derived neurotrophic factor (BDNF) were analyzed. The group that migrated to an altitude of 4500 m presented significant associations between mood and cognitive functions, including reaction time, memory and attention. Finally, a recent meta-analysis showed that hypoxia harms cognition [34]

Regardless of the altitude, hypoxia level and exposure time, the literature showed the effects of this environmental condition on complex cognitive capabilities. However, the mechanisms that can modulate the cognitive deterioration in hypoxia are not yet fully known.

It is possible that intestinal changes negatively affect the central nervous system via the gut-brain axis, and that changes in the gut microbiota because of hypoxia. Models of hypobaric hypoxia, similar to high altitude, appear to affect the intestinal barrier mechanism, increasing intestinal permeability [35]. Other hypoxia models, such as intermittent hypoxia, common in sleep apnea, also appear to affect the intestinal microbiota [36]. Zhang et al. (2018) found that animals submitted to hypoxia have changes in the gut microbiota, including a relative abundance of class *Epsilonproteobacteria, phylum Actinobacteria* and class *Erysipelotrichia* significantly decreased. In addition, at the genus level, the relative abundance of *Helicobacter* was decreased, while the relative abundance of genus *Alistipes* increased in the hypoxia group [37].

## 5. Inflammation, Cognition and Mood

The brain structures responsible for cognitive and mood impairment are interconnected. One hypothesis for the interaction between cognition, mood and the brain under stress conditions is the release of glucocorticoids through the hypothalamic–pituitary–adrenal (HPA) axis compromises the hippocampus concerning neuronal survival [38,39]. The hippocampus is one of the most connected brain areas, beginning to emerge as an integrator of emotion and cognition [40]. The compromised hippocampus can negatively affect learning [41] and mood [42].

In situations that disrupt the integrity of the organism, such as tissue injury, which can occur in hypoxia, many metabolic and systemic alterations aim to re-establish homeostasis, initiating a cascade of acute response events. Cytokines such as IL-1 and TNF-α stimulate the hepatic production of C-reactive protein (CRP), which is considered the main acute phase protein during inflammatory responses. Among the functions attributed to CRP, the ability to bind to the cell membrane components allows tissue repair, inhibition of tumor cell growth, modulation of polymorphonuclear and monocyte cell function, aggregation and platelet secretion are included [43].

Elevated CRP is associated with neurocognitive and mood state decline [44]. It is possible that a reduction in the BBB integrity, mediated by the inflammatory process, promotes more vulnerable CNS to infiltration periphery molecules [45].

The association between CRP levels and cognitive performance in patients during the acute phase of psychosis was recently investigated [46]. Sixty-two patients were tested at discharge or after six weeks with CRP measurements and alternative neuropsychological assessment forms. The results showed an inverse correlation between cognitive performance and increased CRP levels. Older adults with bipolar disorder were divided into bipolar (*n* = 21) and control groups (*n* = 26). The serum IL-1 concentration was elevated in bipolar subjects and associated with volunteers who presented worsening cognitive function [47].

Hypoxia can cause the translocation of NF-kB to the nucleus in blood mononuclear cells, and this translocation may occur due to stimuli such as neurotransmitters (glutamate) and an increase in pro-inflammatory cytokines (IL-1 and TNF-α) [48]. Through the NF-kB, it is possible to observe the beginning of several neurodegenerative processes associated with worsening memory and learning [49,50]. Using a culture of cells from healthy volunteers in a hypoxia condition (1% O_2_), the synthesis of TNF-α from monocytes stimulated by lipopolysaccharide (LPS) was higher [51].

In one of the few studies analyzing altitude, inflammation and cognition, it was possible to verify a correlation between the increase in CRP levels and cognitive impairment. In total, 100 militaries climbed to an altitude of 3650 m, and, after seven days, climbed to an altitude equivalent to 4400 m, where they remained for three months [44]. According to Goldstein et al. (2014), CRP induces cognitive impairment may involve cytotoxicity and structural brain changes from inflammation. Jefferson et al. (2007) showed an association between smaller brain volumes as measured via magnetic resonance imaging and higher CRP levels. CRP and cognition-related tests measured at sea level, 3650 m, 4400 m and sea level after one month of climbing. After one month, the CRP concentration was restored to basal values.

Cognitive function, BDNF, IL-1 and vascular endothelial growth factor (VEGF) at an altitude of 3900 m analyzed in 105 trained and sedentary volunteers. Both groups demonstrated significantly better cognitive performances at sea level than at altitude [22]. One of the hypotheses for these results was that BDNF decreased, and IL-1 increased during hypoxia. Hypoxia induces mood changes mediated by inflammation and increased IL-6, CRP and other inflammatory markers that may impair mood [52], similarly to increased IL-6, IL-8 and TNF-α in individuals with a mood disorder such as bipolar and depression [53,54].

## 6. Glutamine, Inflammation, Mood and Cognition

Whereas hypoxia generates an inflammatory state, we propose that the scenario can impair mood and cognition. The glutamine supplementation may be a potential attenuating agent of cognitive and mood worsens mediated by high altitude inflammation. Although strategies to prevent or mitigate the worse psychobiological because hypoxia effects are relevant, most studies evaluate the dietary or nutritional effect on mood only during or after physical exercise [55,56,57,58,59,60,61,62,63,64,65].

Glutamine is a nonessential amino acid (AA) that may be synthesized by the body. It is the most abundant AA in plasma and muscle tissue and metabolized in the gut, liver and kidney. Glutamine performs numerous functions, including in the immune system, acid–base balance, transport of ammonia, carbon skeleton donation for gluconeogenesis, nucleotide synthesis, induction of gene expression of a wide variety of proteins and synthesis of neurotransmitters such as glutamate and γ-aminobutyric acid (GABA) [66]. In hypoxia, the glutamine synthesis may not be sufficient to meet the increased demand. Elite runners exposed to a moderate altitude of 1640 m for four weeks presented reduced serum glutamine concomitantly with an increase in the incidence of upper respiratory tract infections (URTI) [7].

Studies have shown that a single dose of glutamine may not be sufficient to substantially increase the plasma concentration of this amino acid since a considerable part of the glutamine consumed orally may be uptake by enterocytes [6,67,68]. Long-term glutamine supplementation increases glutamine levels in several tissues, such as the liver, brain and immune cells [69,70,71,72]. This is one of the justifications for its use for periods higher than one day in preoperative cases. It has also suggested that night is the best period for glutamine supplementation since it is the highest immune cell activity [73].

Some tissues can synthesize and use glutamine, whereas others, such as the brain and kidneys, cannot synthesize glutamine due to the absence of the enzyme glutamine synthetase. Thus, it is possible to consider that some tissues are only consumers of glutamine since they depend on serum level availability. The variations between plasma and tissue glutamine are since small amounts of free glutamine in the body are in the blood. Even in situations of less glutamine availability (±400 µM), for example, some cells remain functioning (i.e., lymphocytes), while others (i.e., macrophages) appear to be less efficient with less glutamine availability (<600 µM) [74,75]. Checking the relationship between free glutamine in the blood and brain is essential to speculate the potential positive effects of supplementation on glutamine intracerebral concentrations. Although it is possible to measure glutamine in the CNS by magnetic resonance, this is not a simple, cheap and accessible technique. Recently, Takado et al. (2019) found that blood glutamine level is positively associated with glutamine levels in the posterior cingulate cortex, cerebellum (r = 0.72; *p* < 0.025). It is possible that the glutamine supplementation, beyond the adequate glutamine amount obtaining by the diet, can significantly increase the levels of glutamine in the blood and the brain.

Several animal and human studies have demonstrated glutamine ability to attenuate inflammatory mediators released in injury and stress conditions. Few studies have evaluated the effects of glutamine supplementation on hypoxia, mood and cognition [8,9,76,77,78,79]. Therefore, we intend to demonstrate the influence of glutamine supplementation on inflammation and subsequently concerning these two psychobiological factors.

When evaluating the effect of glutamine supplementation on cytokine release, organ damage and survival of endotoxin-induced septic shock, a single dose of glutamine (0.75 g/kg) significantly attenuated TNF-α release for 2 h and IL-1-β for 4 h. These data indicate that glutamine can attenuate the release of pro-inflammatory cytokines and protect against tissue damage [80]. Glutamine can mitigate inflammation, mainly by reducing the leakage of substances from the gut. These findings studies in humans seem to corroborate, suggesting that glutamine contributes to a better gut environment [81].

Our laboratory evaluated the effects of glutamine supplementation on immune response regulation mediated by lymphocytes and inflammation in humans exposed to 6 h of simulated hypoxia at 4500 m in a normobaric chamber, with and without exercise. Before hypoxia, the volunteers received 20 g of glutamine per day (high dose) during the night for six days. We verify that glutamine supplementation stimulated both T helper 1 lymphocyte response, inhibiting T helper 2 lymphocyte response and mitigate IL-6 and TNF-α release [9].

In a hypoxic condition, synaptic signaling decreases as the oxidative stress and inflammation increase. HIF-1α is upregulated and increases pro-inflammatory cytokines, such as IL-β, IL-6, IL-8 and TNF-α, leading to activation of the NF-κB signaling pathway, possibly affecting neurotransmitter functioning. Thus, inflammation is associated with behavioral changes and mood disturbances [82,83]. Still, hypoxia can increase damage-associated molecular patterns (DAMPs) that can bind to cellular receptors, such as TLR-4 in microglia and activate inflammatory pathways [84]. There is evidence that hypoxia may adversely affect gut microbiota diversity and increase inflammation [36,85].

The gut–brain axis can contribute to changes in the central nervous system [86,87,88]. It is possible that glutamine improves the intestinal environment and reduces inflammation mediated by leak gut, and, thus, improves cognition and mood [89].

Akisu et al. (2003) analyzed the influence of glutamine supplementation on the inflammatory mediator release and intestinal lesions in hypoxia. Young mice were divided into four groups: hypoxia, hypoxia with glutamine supplementation (0.5 g/dL) for three days; hypoxia with glutamine supplementation (3 g/dL) for ten days; and normoxia. The results showed that hypoxia with the glutamine supplementation group presented attenuated gastrointestinal damage and decreased TNF-α levels compared to other groups [90]. The authors concluded that glutamine reduces the lesions induced by hypoxia by inhibiting the release of intestinal cytokines. This benefit can be attributed to glutamine potential effects on the intestinal barrier mechanism and decreasing harmful substances translocation [80]. Recently, the effects of glutamine supplementation on pro-inflammatory cytokines and pro/anti-inflammatory balance were investigated in rats with severe acute pancreatitis. Eighty animals were divided into 5 groups: control; enteral nutrition; parenteral nutrition (glucose, amino acid compound, fat and water-soluble vitamin); enteral nutrition + glutamine (0.4 g/kg); and parenteral nutrition + glutamine (0.4 g/kg). The combination of enteral nutrition and glutamine effectively improved inflammation and regulated the pro/anti-inflammatory balance [91].

Glutamine supplementation seems to benefit gut microbiota diversity, which may reduce inflammation. The gut–brain axis could also justify the possible benefits of glutamine and cognitive aspects [81]. The relationship between gut microbiota, inflammation and cerebral health seems to have gained considerable notoriety in recent years [92]. Glutamine supplementation can attenuate oxidative stress and inflammation and may attenuate neurotransmission disturbance [93]. Additionally, the hypoxia condition affects the glutamine–glutamate cycle in the CNS, contributing to neurotransmission changes. Glutamine could affect neurotransmission since the significant role in the brain is that of a precursor of neurotransmitter amino acids: the excitatory amino acids, glutamate (Glu) and aspartate (Asp) and the inhibitory amino acid, GABA [94].

**Figure 1:**
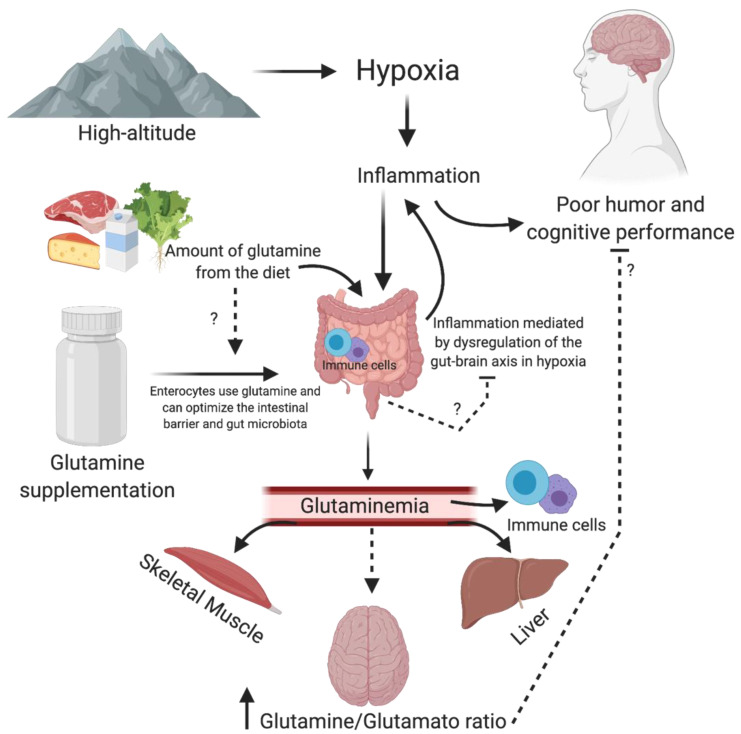
Possible mechanism of action of glutamine on cognition and mood. Hypoxia from high altitude, through inflammation, can affect neurotransmitters activity in the brain, worsening mood and cognition. Moreover, hypoxia can negatively affect the gut–brain axis by dysregulation of gut microbiota and or immune cells in the gut. The increase in the glutamine–glutamate ratio favors excitatory activity, which may improve mood and cognition. Moreover, glutamine could improve the intestinal barrier by immune cells and balance the gut microbiota.

Moreover, a few studies have suggested that glutamine, per se, may have a central action modulating neurotransmitter synthesis and consequently modulating cognitive and mood aspects. For example, in 1993, Young et al. submitted marrow transplantation patients (*n* = 23) to parenteral nutrition (control group) versus glutamine supplementation (40 g) plus parenteral nutrition (intervention group). The solutions were isocaloric and administered until the patient was eating 50% of estimated requirements. The authors verified improvement in the mood analyzed by the profile mood states questionnaire (POMS) [95]. This study was conducted with unhealthy patients and was not a hypoxic condition. However, it suggests the relationship between glutamine and mood. Arwert et al. (2003) verify that glutamine supplementation (5 g) in 14 men and 28 women with severe psychiatric histories were effective for improving short-term memory, concentration, attention and vigor compared to the placebo group. These changes were associated with the release of IGF-1 [79]. Glutamine is also essential for children. Low-weight preterm infants supplemented with glutamine (0.3 g/kg) from the 3rd to 30th day of life. Later, at the age of seven, the same volunteers underwent motor and cognitive tests. The researchers observed beneficial effects in attention, working memory, information processing, reaction time and motor tests in the supplemented children [78]. A possible mechanism of action of glutamine on cognition and mood is described in Figure 1.

More recently, Strasser et al. (2020) found that the glutamine–glutamate ratio in the nucleus accumbens affects human cognitive performance. Twenty-seven subjects (20–30 years old) were evaluated for glutamate, glutamine and GABA concentration and their ratios (glutamine/glutamate and GABA/glutamine) using magnetic resonance. The authors found that the resting state acumbal glutamine-to-glutamate ratio predicts an effort-based motivated task overall average performance and the higher levels of glutamine were positively associated with the success rate of the proposed test (r = 0.46; *p* < 0.05) and negatively with the perception of effort (r = −0.42; *p* < 0.05). The same can be observed by evaluating the glutamine/glutamate ratio, positive (r = 0.46; *p* < 0.05) and negative (r = −0.49; *p* < 0.05) association for success rate and perceived exertion, respectively. The potential positive effect of glutamine on mood and cognition can be associated with its increase in brain areas and its effect on inflammatory control.

It is common, however, for people to believe that glutamine is free from adverse effects. High brain glutamine levels are positively associated with neuroticism, trait anxiety [96] and hyperammnonemic coma [97]. Many people supplement glutamine because they believe it improves the immune system, memory and cognition, even for those who do not exercise. Glutamine supplementation generally ranges from 2–40 g/day (high dose of glutamine) [98]. Longitudinal studies that evaluated supplementation in large doses are scarce, and it is challenging to infer more specific damage. Diets rich in glutamine could alter amino acid transport across the cell membrane, increase glutamine metabolites’ production due to enhanced breakdown of glutamine and accumulation of glutamine precursors due to the impaired endogenous synthesis of glutamine, alterations in amino acid concentrations in body fluids and others. In the brain, in particular, increased glutamine levels can affect the brain glutamine–glutamate cycle. Increased glutamine levels in the blood make it difficult for glutamine output in the astrocytes into the bloodstream, affecting ammonia and glutamate levels in the brain [99]. This phenomenon could favor the accumulation of glutamine metabolites in the brain and negatively affect brain function. For example, Oppong et al. (1997) analyzed cirrhotic patients and showed significant impairment in an electroencephalogram during an oral glutamine challenge [100]. However, besides this study being with sick patients, few studies have checked the effect of chronic glutamine consumption (in high doses) on the brain’s adverse outcomes. Despite speculation about the positive (anti-inflammatory) effects of glutamine to consider potential adverse effects is essential.

Finally, despite our hypotheses about the positive effects of glutamine supplementation on cognition and mood, little is discussed about the interaction between glutamine obtained from the diet. There is an increase in demand under different conditions. Specific dietary patterns low in glutamine may benefit more, while those with higher amounts of glutamine in the diet are less benefited. Lenders et al. (2009) showed that glutamine (6.85 ± 2.19 g/day), glutamate (7.27 ± 2.44 g/day), accounts for the highest intake in protein-based diets [101]. Thus, considering the triad: habitual glutamine intake, body demand in different situations (health, illness, physical exercise and high altitude), and supplementation is crucial to understand the effects of glutamine in the short, medium and long term.

## 7. Conclusions and Limitations

We suggest that mood and cognition worsening induced by inflammation in hypoxia may be attenuated by glutamine supplementation. However, randomized placebo-controlled clinical studies must be carried out with high methodological rigor (internal validity) to refute or endorse this hypothesis. Considering the complexity of hypoxia and the countless people affected by its harm, strategies that contribute to this situation should be encouraged. This study is limited as there are still few studies on this topic, and assessing the cerebral concentration of glutamine is difficult. Studies in hypoxia, with and without glutamine supplementation, should be performed to experimentally correlate the relationship between glutamine and cognitive function and mood in hypoxia.

## Figures and Tables

**Table 1:** Search strategy.

	MeSH, Entry Terms and Keywords Combinations Used to Search the Articles.
#1	Hypoxia OR High Altitude OR Altitude Sickness OR Mountain Sickness OR Simulated Altitude AND Mood disorders
#2	Hypoxia OR High Altitude OR Altitude Sickness OR Mountain Sickness OR Simulated Altitude AND Cognition disorders
#3	Hypoxia OR High Altitude OR Altitude Sickness OR Mountain Sickness OR Simulated Altitude AND inflammation OR neurogenic inflammation
#4	Hypoxia OR High Altitude OR Altitude Sickness OR Mountain Sickness OR Simulated Altitude AND Glutamine OR L-Glutamine OR L Glutamine
#5	Hypoxia OR High Altitude OR Altitude Sickness OR Mountain Sickness OR Simulated Altitude AND Cognition OR Cognition disorders AND Affect OR Mood disorders AND inflammation OR neurogenic inflammation
#6	Hypoxia OR High Altitude OR Altitude Sickness OR Mountain Sickness OR Simulated Altitude AND Cognition OR Cognition disorders AND Affect OR Mood disorders AND inflammation OR neurogenic inflammation AND OR L-Glutamine OR L Glutamine

Legend: terms searched on MEDLINE/PubMed (http://www.ncbi.nlm.nih.gov/pubmed) on 4 November 2020.
